# Coexistence of pulmonary sclerosing hemangioma and primary adenocarcinoma in the same nodule of lung

**DOI:** 10.1186/1746-1596-6-41

**Published:** 2011-05-20

**Authors:** Wei Liu, Xiao-Ying Tian, Yang Li, Yong Zhao, Bin Li, Zhi Li

**Affiliations:** 1 Department of Pathology, The First Affiliated Hospital, Sun Yat-sen University. 58, Zhongshan Road II, Guangzhou 510080, China; 2 Department of Pathology, Longgang District Central Hospital of Shenzhen. 1228, Shenhui Road, Shenzhen 518116, China; 3 Centre for Cancer and Inflammation Research, School of Chinese Medicine, Hong Kong Baptist University. 7, Baptist University Road, Kowloon Tong, Hong Kong, China

## Abstract

Pulmonary sclerosing hemangiomas (PSH) of the lung are uncommon tumors and may present cytological atypia with unusual manifestations. The development of PSH combined with other different tumors in lung is extremely rare. We report a case of coexistence of PSH and primary adenocarcinoma in a young female occurring in the same pulmonary nodular mass of right lower lobe. The solitary mass of lung was well-circumscribed on chest computed tomography (CT) and gross examination. Histologically, the mass contained two separated portions and displayed typically histological features of PSH and acinar adenocarcinoma, respectively. In PSH portion, the tumor was composed of sheets of round cells with scattered surface cuboidal cells forming small tubules. Both round and surface cells were diffusely positive for epithelial membrane antigen (EMA) and thyroid transcription factor-1 (TTF-1), but lack immunoreactivity for pancytokeratin in round cells. In adenocarcinoma portion, the tumor cells formed irregular-shaped glands with cytologically malignant cells infiltrating in fibroblastic stroma, and no TTF-1-positive round cells could be observed in this portion. Under the microscopy, there was no gradual transition of these two portions observed in mass. A diagnosis of PSH combined with primary adenocarcinoma of lung was made. There was no evidence of tumor recurrence during the period of postoperative 6-month follow-up. To our knowledge, this is the first case of coexistence of PSH and adenocarcinoma in the same nodule of lung. In addition, the biological behavior and histological differential diagnosis of this tumor were also discussed.

## Background

Pulmonary sclerosing hemangioma (PSH) is an uncommon tumor and is thought to be a benign neoplasm of lung [1,2]. However, histogenesis and biological behavior of this tumor has not yet been clarified adequately. Histologically, the characteristics of PSH have been well-described as papillary, solid, sclerotic, or hemorrhagic patterns. PSH comprises two well-accepted cell types: eosinophilic cuboidal epithelial lining cells and round cells with either eosinophilic or clear cytoplasm [2]. Although most PSH cases have a mixture of these four histological patterns with two cell types, unusual morphological presentations have been revealed in a few cases, such as atypical adenomatous hyperplasia or adenocarcinoma-like area within the PSH [3]. However, to our knowledge, so far there is no report to describe a coexistence of PSH and primary adenocarcinoma within the same lobe of lung in English literatures. Herein we report a young female with PSH combined with adenocarcinoma in the same nodule of right lower lobe. Histopathological findings and imaging features, as well as outcome and differential diagnosis are to be discussed.

## Case presentation

**Figure 1:**
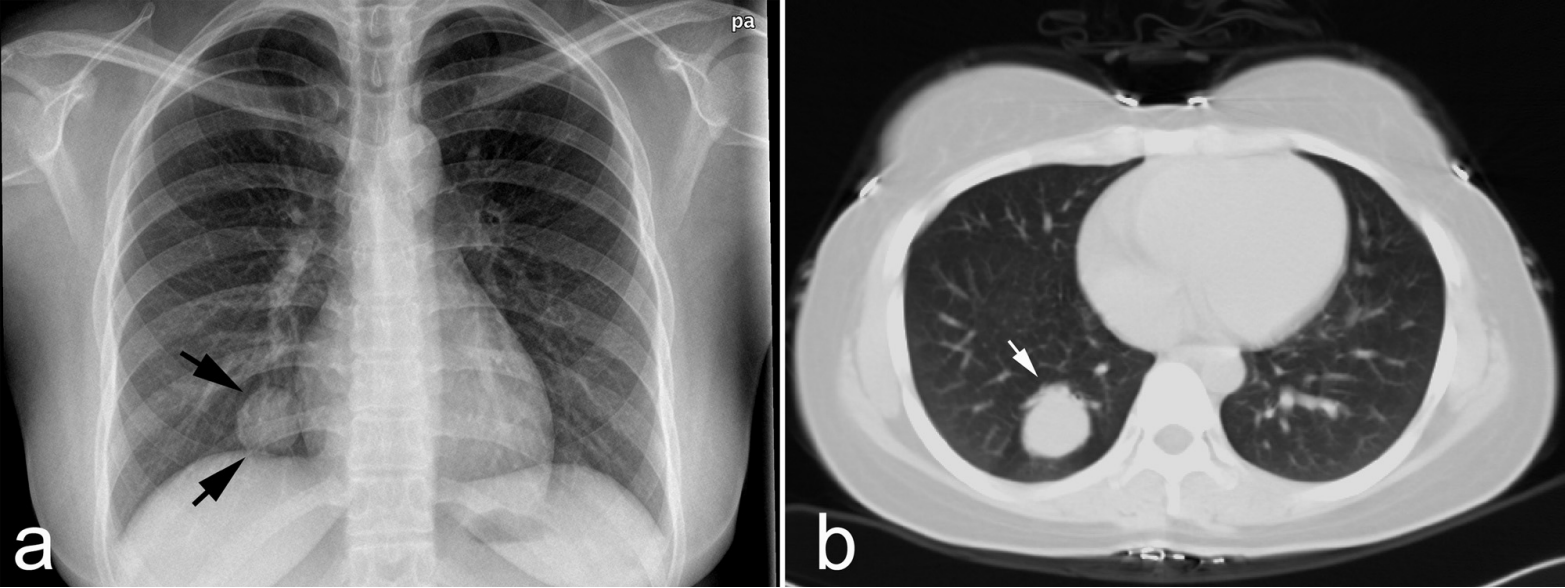
**Preoperative chest radiology of lesion**. (a) Chest X-ray revealed solitary pulmonary mass in right lower lung field (black arrow). (b) Chest CT image showed well-defined mass in right lower lobe (white arrow). There was no remarkable image finding was noted in the surrounding lung parenchyma.

**Figure 2:**
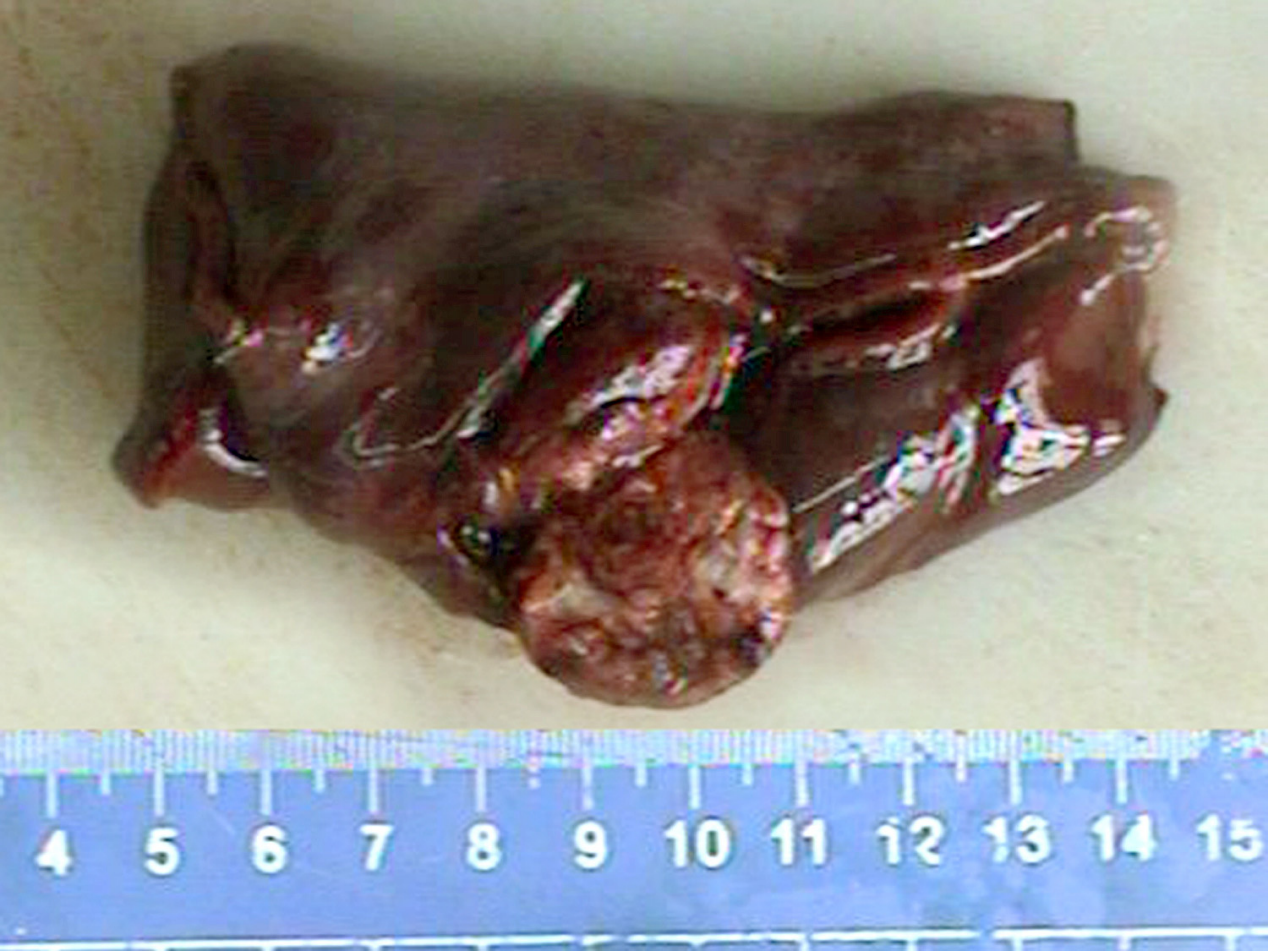
**Postoperative gross examination of lesion**. On macroscopical examination, the lesion was gray-red solitary nodular mass without gross necrosis, haemorrhage and calcification, measuring 2.7 × 2.5 cm, was located in the lung parechyma. The mass was well-circumscribed, but there was no fibrous capsule round the mass.

A 22-year-old woman with a 4-month history of pulmonary mass was admitted to our hospital for further examination and treatment. The pulmonary mass was first detected on chest radiography when she underwent annual body examination at a local hospital (Figure 1A). For unknown reasons, the patient failed to receive workup and management at that time. On admission in our hospital, physical examination and routine laboratory test was no positive finding. Sputum examination showed no pathogenic organisms and malignant cells. Chest computed tomography (CT) revealed a well-defined 2.5 cm mass with CT attenuation value of 35 HU was located in the right lower lobe of lung. Contrast-enhanced CT was not performed. Neither enlarged lymph node nor remarkable image finding was noted in the surrounding lung parenchyma (Figure 1B). According to the recommendation of CT examination, the patient underwent fiberoptic bronchoscopy and transbronchial biopsy, but there was no definitive diagnosis obtained by histological examination. A preoperative presumed diagnosis was PSH and inflammatory pseudotumor of lung. A partial segmentextomy of the right lower lobe was performed, and macroscopical examination revealed a gray-red solitary nodular mass, measuring 2.7 × 2.5 cm, located in the lung parechyma. The mass was well-circumscribed, but there was no fibrous capsule around the mass (Figure 2).

**Figure 3:**
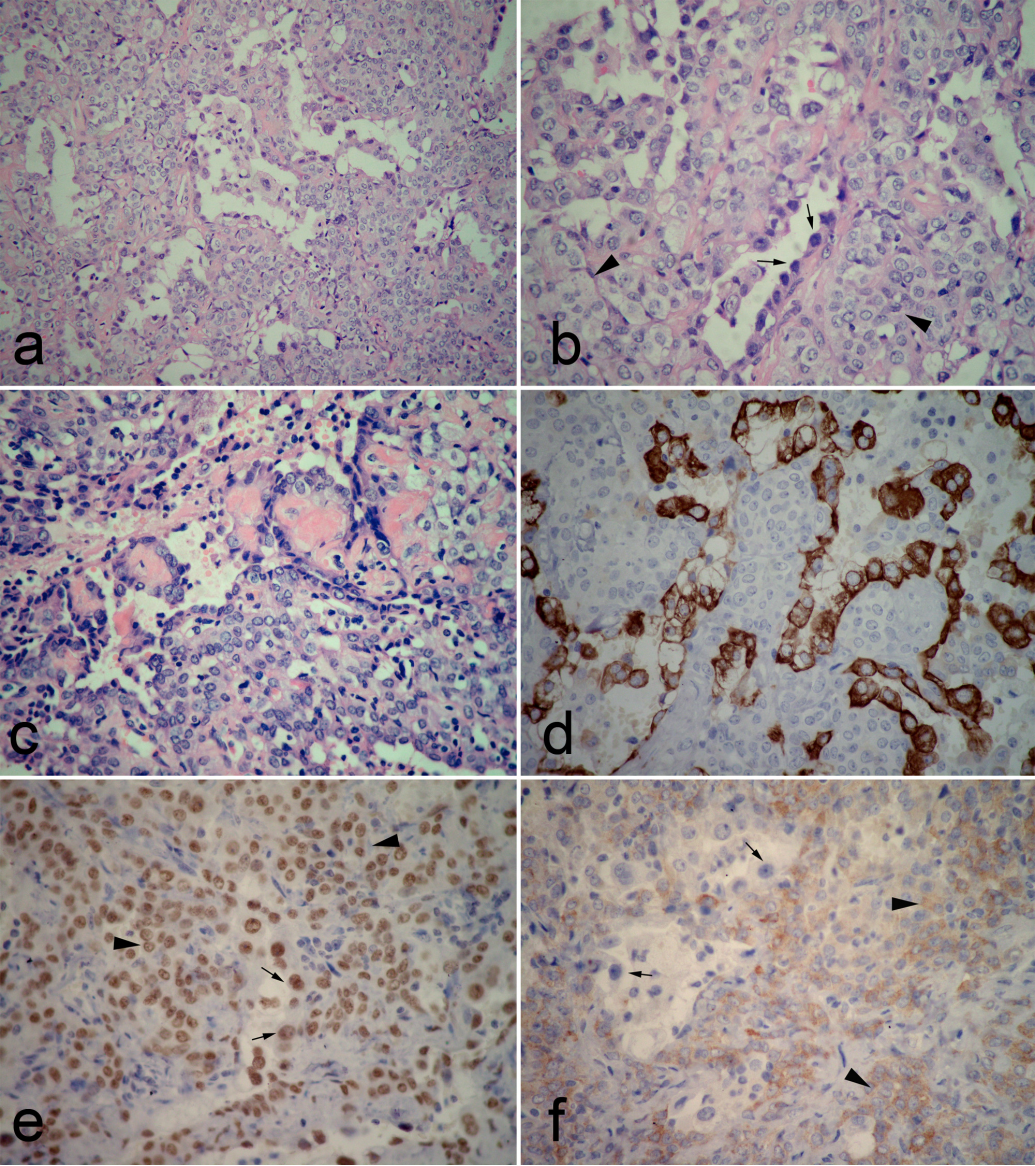
**Postoperative photomicrographs of lesion in PSH portion**. (a) the tumor was composed of sheets of round cells with scattered surface cuboidal cells forming small tubules. (b) The surface cuboidal cells demonstrated vacuolated and foamy cytoplasm with focal mild nuclear atypia (black arrow). The round cells showed uniform, medium-size polygonal nuclei with moderate amounts of pale, eosinophilic or clear cytoplasm (black arrowhead). (c) In some area, papillary pattern with hyalinizing stalks could be obsreved. Both surface cuboidal cells and round cells could be found in this structure. (d) Immunohistochemically, positivity of surface cuboidal cells, but negativity of round cells for pancytokeratin was found in tumor. (e) Both surface (black arrow) and round cells (black arrowhead) showed strongly positive for TTF-1. (f) A focal weak positivity of round cells (black arrowhead) for synaptophysin was observed in this portion, but the surface cells (black arrow) showed negative for Syn. (a, H&E staining with original magnification of 200 ×; b-c, H&E staining with original magnification of 400 ×; d-f, immunohistochemical staining with original magnification of 400 ×).

**Figure 4:**
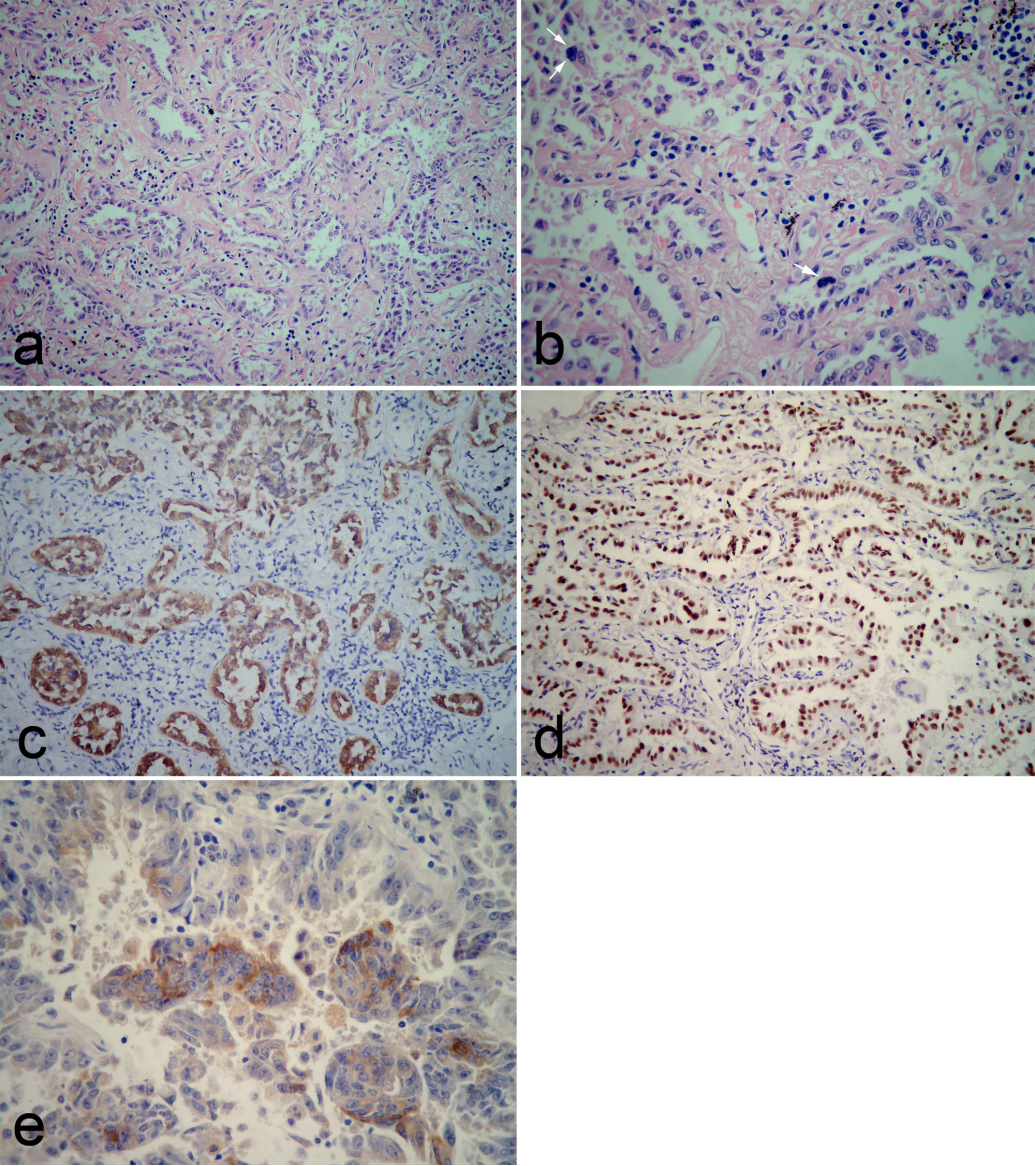
**Postoperative photomicrographs of lesion in adenocarcinoma portion**. (a) The tumor cells formed irregular-shaped glands with infiltrating in the fibroblastic stroma and no gradual transition between adenocarcinoma and PSH was observed. (b) The tumor cells exhibited cytologically hyperchromatic nuclei, obvious nucleoli and mitotic figures (white arrow). There was no round cells found to distributed in the interstitial portion of gland structures. Immunohistochemically, tumor cells were positive for pancytokeratin (c), TTF-1 (d) and CEA (e). There was no TTF-1-positive round cell observed in this portion of mass (a, H&E staining with original magnification of 200 ×; b, H&E staining with original magnification of 400 ×; c-d, immunohistochemical staining with original magnification of 200 ×; e, immunohistochemical staining with original magnification of 400 ×).

Under the microscopical examination, part of nodular mass, measuring approximately 1.2 cm in longest diameter, demonstrated classical solid pattern of PSH with varying proportions of surface cuboidal cells and pale round cells. In this area, the mass was composed of sheets of round cells with scattered surface cuboidal cells forming small tubules (Figure 3A). The surface cuboidal cells resembling type II alveolar cells demonstrated vacuolated and foamy cytoplasm with focal mild nuclear atypia. While the round cells were pale round cells showed uniform, medium-size polygonal nuclei with moderate amounts of pale, eosinophilic or clear cytoplasm (Figure 3B). In some area, papillary pattern with hyalinizing stalks was also obsreved, and the surface cuboidal cells lined the papillae surface (Figure 3C). Neither necrosis nor mitotic activity was found in PSH area. Immunohistochemically, round cells were positive to epithelial membrane antigen (EMA), thyroid transcription factor-1 (TTF-1), Estrogen receptor (ER), progesterone receptor (PR), and focally weak positive for synaptophysin (Syn), but negative for pancytokeractin (AE1/AE3). Surface cells were strongly positive for AE1/AE3, EMA and TTF-1, but negative for ER, PR, Syn and carcinoembryonic antigen (CEA) (Figure 3D-F). Interestingly, however, acinar adenocarcinoma could be observed in other area of the same nodular mass. In this area, measuring approximately 1.0 cm in longest diameter, the tumor cells formed irregular-shaped glands with cytologically malignant cells exhibiting hyperchromatic nuclei in a fibroblastic stroma (Figure 4A). There was no round cells found to distribute in the interstitial portion of gland structures (Figure 4B). The field of PSH and acinar adenocarcinoma was separated in the mass, and there was no gradual transition of these two parts observed in mass. Immunohistochemically, tumor cells of adenocarcinoma were positive for AE1/AE3, EMA, TTF-1 and CEA, but negative for ER, PR, and Syn (Figure 4C-E). In addition, no TTF-1-positive stromal cells could be observed in adenocarcinoma portion of mass by immunohistochemical staining. Based on above findings, a final histological diagnosis of PSH combined with primary adenocarcinoma was made. After surgery, 6 months follow-up was performed without further treatment. There was no evidence of tumor recurrence during the period of postoperative follow-up.

The term of "pulmonary sclerosing hemangioma (PSH)" was first used in 1956 by Liebow and Hubbell, in which sclerosing hemangioma was described as a variant of hemangioma due to prominent angiomatoid features and occurred predominantly in young or middle-aged women [1]. In 1981, WHO classification of lung tumors accepted PSH as a tumor-like lesion [4]. However, the histogenesis of PSH has not yet been elucidated since it first description. Subsequent studies have postulated that PSH might originate from mesothelium [5], mesenchyma [6], epithelium [7], and neuroendocrine cells [8]. Recent immunohistochemical studies revealed that PSH might derive from primitive undifferentiated respiratory epithelium [9,10], and molecular study has also demonstrated the same monoclonal pattern in both the round and surface cells, consistent with a ture neoplasm rather than a hamartoma [11]. Therefore, the PSH was categorized as a miscellaneous tumor of lung in 1999 and 2004 WHO classification of lung tumors [2,12].

In the current study, we found that both surface and round cells in PSH portion of mass were immuno-positive for TTF-1 and EMA, but round cells were negative for pancytokeratin. These results provided useful clues not only for histogenesis but also for the diagnosis of this tumor. However, in contrast to most previous studies, the focal weak positivity of round cells, but not surface cells, for neuroendocrine marker, synaptophysin (Syn) was found in our case. This result indicated that tumor cells in PSH, in particular round cells, might have potential to neuroendocrine differentiation or represent incompletely differentiated Clara cells. It is well known that TTF-1 expressed in the non-ciliated columnar cells of the fetal lung as early as 11 weeks of gestation, and in type II pneumocytes and Clara cells of the adult lung [13,14], and both surface cells and round cells of PSH represented a variable differentiation from a common progenitor cell [11]. In the present case, the immunohistochemical positivity of round cells for TTF-1 and Syn supported that these cells were derived from a primitive respiratory cell, and part of round cells might be intermediate differentiation between undifferentiated primitive respiratory cell and differentiated Clara cells or pneumocytes [9,10]. That might be the reason why typical carcinoid could be generated from PSH [15]. Of course, larger series should be performed to verify this postulation.

PSH is a distinctive lung tumor, which is composed of mixtures of papillary, solid, sclerotic, and/or hemorrhagic patterns with varying proportions of two cell types. Variable degrees of cytologic atypia were found in most PSH. The cases of atypical alveolar hyperplasia (AAH) in PSH [16], alveolar adenoma with PSH [17], and transition from atypical hyperplasia of type II pneumocyte to PSH [18] have been described in literatures. Recently, a case of alveolar adenoma-like area in the peripheral portion of the PSH, and a case of papillary adenocarcinoma-like area within the PSH have also been reported [3]. These findings suggested that AAH of type II pneumocytes could be a focus of atypical growth of lining epithelial cells of PSH, or AAH- and adenocarcinoma-like lesion with papillae might be presumptive precursor lesion of PSH. However, in contrast to previous studies, in our case we found acinar adenocarcinoma coexisted with PSH in the same nodular mass. We wondered if this adenocarcinoma portion of mass was a substantive primary tumor or just a subordinate adenocarcinoma-like area within the PSH. We reviewed the histological and immunohistochemical features of unusual presentations within the PSH in securable literatures. We found that (a) a gradual transition from unusual structures, including AAH, avleolar adenoma-like area and adenocarcinoma-like area, to the majority of PSH could be observed in all reported cases; (b) TTF-1 positive round cells could be detected in varied amount in the interstitial portion of unusual structures. However, in the present case, adenocarcinoma portion and PSH portion were separated distribution without gradual transition area under the microscopical examination by serial section, and there was no TTF-1 positive round cells found in the fibroblastic stroma of adenocarcinoma. Based on those findings, we considered the adenocarcinoma portion was a true primary tumor rather than an unusual histological presentation of PSH. Existence of adenocarcinoma and PSH in different segmental nodular mass of pulmonary lobe has been described [19]. But to our knowledge, ours is the first case to demonstrate the coexistence of adenocarcinoma and PSH within the same nodule of lung.

PSH is a clinically benign tumor and has a good prognosis after surgical resection. However, lymph node metastasis and multifocal distribution of PSH have been found in a few cases [3,15,16,20,21]. Moreover, moderate to severe cytological atypia, and sometimes combined with unusual structures, might make PSH like a malignancy. The main histological differential diagnosis includes papillary alveolar adenoma, bronchioloalveolar carcinoma, carcinoid, and papillary adenocarcinoma, all of which exhibit papillary, solid or tubular appearance and may sometimes cause diagnostic confusion with PSH, especially in insufficient biopsy specimen. However, the papillary-like structure in PSH is not true papillae, which is composed of lining cuboidal cells and round cells below rather than fibrovascular cores present in other tumors with true papillary structure. The presence of pale round cells in tumor and immuno-positivity of round cells for TTF-1, EMA and ER, PR support the diagnosis of PSH. There are histological similarities between carcinoid and PSH when PSH displays solid histological pattern predominantly, in which uniform round cells are arranged in sheets pattern. But lack of strong diffuse TTF-1 reactivity may also be useful in excluding carcinoid. Together with histological features, immunohistochemical evaluation with a panel of EMA, pancytokeratin and TTF-1 is most useful in distinguishing PSH from other similar tumors.

## Conclusion

We herein first reported a rare case of PSH combined with primary adenocarcinoma occurred in the same nodular mass of lung. The mass contained two separated portions and displayed typically histological features of PSH and adenocarcinoma, respectively. The precise mechanism of coexistence of PSH and adenocarcinoma in the same nodule remains unknown, and longer period of follow-up and more case investigation are necessary to better clarify the biological characteristics and clinical outcomes of this unusual tumor.

## Consent

Written informed consent was obtained from the patient for publication of this case report and any accompanying images. A copy of the written consent is available for review by the Editor-in-Chief of this journal.

## Competing interests

The authors declare that they have no competing interests.

## Authors' contributions

LIU W and LI Y made contributions to acquisition of clinical data, and analysis the histological features of case by H&E staining. TIAN XY drafted the manuscript. LI Z revised manuscript critically for important intellectual content and has given final approval of the version to be published. ZHAO Y and LI B carried out the immunoassays. All authors read and approved the final manuscript.
